# Monotone Quantifiers Emerge via Iterated Learning

**DOI:** 10.1111/cogs.13027

**Published:** 2021-08-11

**Authors:** Fausto Carcassi, Shane Steinert‐Threlkeld, Jakub Szymanik

**Affiliations:** ^1^ Department of Linguistics University of Amsterdam; ^2^ Department of Linguistics University of Washington

**Keywords:** Iterated learning, Generalized quantifiers, Semantic universals, Neural networks, Cultural evolution

## Abstract

Natural languages exhibit many *semantic universals*, that is, properties of meaning shared across all languages. In this paper, we develop an explanation of one very prominent semantic universal, the monotonicity universal. While the existing work has shown that quantifiers satisfying the monotonicity universal are easier to learn, we provide a more complete explanation by considering the emergence of quantifiers from the perspective of cultural evolution. In particular, we show that quantifiers satisfy the monotonicity universal evolve reliably in an iterated learning paradigm with neural networks as agents.

## 1 Introduction

While natural languages show great variability, there are features that they all appear to share. Linguists call these features linguistic *universals*. Universals have been found at all levels of linguistic structure, for example, phonological (Hyman, 2008), syntactic (Newmeyer, 2008), and semantic (Barwise & Cooper, 1981). Some universals might follow from constraints on what humans are physically capable of doing. For instance, there is no language whose prosody requires the production of sound waves above 30 kHz, since humans cannot hear such sounds. The reasons for other universals are harder to understand, leading to multiple proposed explanations.

One well‐supported claim going back at least to Chomsky (1969) is that at least some universals are to be explained in terms of *learnability*. According to one version of this approach, it is easier to learn a language that satisfies the universal than to learn a language that does not satisfy the universal, and this difference in the complexity of acquisition produces, through processes of cultural evolution, languages that satisfy the universals. This picture of the emergence of universals has been supported by the previous computational and experimental work in the domains of semantics (Piantadosi, Tenenbaum, & Goodman, 2013; Steinert‐Threlkeld & Szymanik, 2019, for example), morphology (Culbertson & Kirby, 2016), phonology (Hayes, Kirchner, & Steriade, 2004; Martin & Peperkamp, 2020; Moreton, 2008; Wilson, 2006) among others. In the case of universals of lexical semantics such as the one we focus on next, the learnability explanation for a universal says that the universal holds because meanings that satisfy the universal are easier to acquire, and therefore more likely to be lexicalized.^1^ Complicated meanings can be obtained through complex grammatical constructions and compositional interpretation thereof.

The idea that semantic universals are a consequence of learnability is an empirical, causal claim about their origins. One way to support the learnability explanation for a specific semantic universal is to show that for various general models of learning the expressions that satisfy the universal are easier to learn. *Neural networks* offer one such model of learning. The previous work has addressed the learnability challenge by showing that quantifiers, responsive predicates, and color terms that satisfy certain semantic universals are easier to learn than ones that do not for neural networks (Steinert‐Threlkeld, 2019; Steinert‐Threlkeld & Szymanik, 2019, 2020).

Showing that a cognitively grounded model of learning learns more easily meanings that satisfy a universal proves that the universal may have a special role in individual's acquisition. However, learnability is a fact about individual cognition, while a universal is a feature of a whole language. Therefore, a causal, mechanistic picture of the evolution of a universal poses the challenge of connecting these two levels, showing the effects of learnability on emerging language structure. This is the so‐called problem of *linkage* (Kirby, 1999). *Iterated learning* is a method that addresses the problem of linkage. An iterated learning model consists of a series of timesteps, the *generations*. Each generation consists of a population of artificial agents that acquire a language from data produced by the preceding generation. Since learning is a noisy process, the languages of a cultural child and its cultural parent are generally slightly different.^2^ Moreover, the changes introduced in the learning phase are not random, but rather tend to be guided by the child's cognitive biases. As a consequence, over time languages adapt better to the agents' cognitive biases. The crucial insight of iterated learning is then that learning is not an inert process in cultural evolution, but rather guides a population toward languages that better conform to the agents' biases. Ease of learning can, through iterated learning, affect the frequency of different traits (see, e.g., Culbertson and Kirby, 2016; Kirby, Cornish, and Smith, 2008; Tamariz and Kirby, 2016, for discussions of the way individual cognition is reflected in language structure through iterated learning and experimental evidence supporting the connection).

In the context of our research question, the above paragraph means that we now need to connect the neural network models into an iterated chain to see whether, in the case of monotonicity (defined in Section 2), the ease of individual learning can affect the language structure. The previous work has combined iterated learning models with neural network learners. After initial work focused on the emergence of compositionality (Batali, 1998; Kirby & Hurford, 2002; Swarup & Gasser, 2009), there has been a recent surge of interest in the combination of these two models again aimed at explaining compositionality (Chaabouni, Kharitonov, Lazaric, Dupoux, & Baroni, 2019; Cogswell, Lu, Lee, Parikh, & Batra, 2019; Guo et al., 2019; Ren, Guo, Labeau, Cohen, & Kirby, 2020), but the promising combination of iterated learning model and neural networks has not been applied extensively to other problems. In this paper, we apply this combination of methods for the first time to study the evolution of a universal of lexical semantics, more specifically the monotonicity universal for simple determiners.

Determiners are expressions that take a common noun as an argument and return a noun phrase. Determiners can be grammatically simple—for example, *some*, *few*, *most*—or complex—for example, *fewer than three* or *at most five*.^3^ Numerous substantial universals have been identified in the semantics of determiners. In the following, we focus on one of these universals, the universal of *monotonicity* for determiners. We present a computation model of the evolution of the semantic structure of quantifiers. We embed neural networks in an iterated learning model, and show that right monotone quantifiers emerge reliably in the process of cultural evolution. Moreover, we show that this result is robust to variations in the computational model. In particular, we find that the full range of right monotone meanings develops in a variation of the model that also induces the evolution of another universal of quantification, the universal of *quantity*.^4^

The next section briefly reviews the theory of generalized quantification and the semantic universals that have been described in it, with a focus on monotonicity. Then, Section 3 presents the model of agents and cultural evolution, as well as an information‐theoretic measure of the *degree of monotonicity* of a quantifier. The result of this first experiment are presented in Section 3.2. The results show that simple right monotone quantifiers evolve in the setup of model 1. Section 4 presents a variation of the first experiment's model, where children cannot keep track of individual objects. The results, presented in Section 4.2, show that the evolution of right monotonicity is robust to variations in the details of the model. Finally, in Section 5, we discuss possible future directions.

## 2 Quantifiers and right monotonicity

An explanation of the evolution of universals in determiner semantics requires a formal specification of what the space of possible meanings for determiners is. In the semantics literature, determiners are analyzed as expressing generalized quantifiers, that is, properties of sets of subsets of a domain of discourse.^5^ The generalized quantifiers expressed by natural language determiners usually relate exactly two sets A and B, where A is the *left argument* and B the *right argument* of the quantifier, saying whether a certain combination of A and B belongs to the quantifier. These quantifiers can be equivalently understood as taking (the characteristic function of) a set A and returning a function from (the characteristic function of) a set B to truth values. For instance, a combinations of A and B verifies the sentence “most *A*s are *B*” iff the number of As that are B (cardinality of the intersection of A and B, that is, |A∩B|) is greater than the number of As that are not Bs (i.e., |A∖B|), that is,

⟦most⟧={(A,B):|A∩B|>|A∖B|}.

Since any set of pairs of sets defines a quantifier, in a universe with n objects there are $2^{4^{n}}$ many possible quantifiers, a number that soon becomes very large as n increases. Only very few out of this huge collection of possible quantifiers are expressed by simple determiners in any natural language.

As mentioned in the introduction, various universals have been proposed to single out the generalized quantifiers expressed by simple determiners in natural language. One of these universals, which is the focus of this paper, is the *monotonicity* universal presented in Barwise and Cooper (1981), which concerns the determiner's right argument. The monotonicity universal says that all simple determiners (type ⟨1,1⟩) across all languages express quantifiers that are either monotone in the right argument or are conjunctions of quantifiers that are monotone in their right argument.^6^ In practice, in the following we will talk mostly about monotone quantifiers rather than their conjunctions. A quantifier is monotone in its right argument iff it is *upward* monotone in its right argument or *downward* monotone in its right argument. A quantifier Q is upward monotone [downward monotone] in its right argument iff for any three sets A, B, and $B^{\prime }$, if Q(A)(B) and $B\subseteq B^{\prime }$ [$B^{\prime }\subseteq B$] then $Q\left(A\right)\left(B^{\prime }\right)$. As an example, consider the upward right monotone quantifier ⟦most⟧. Assume that the sentence “Most cats sleep” is true and that everything that sleeps is alive, that is, ⟦sleep⟧⊆⟦alive⟧. The fact that ⟦most⟧ is upward monotone in its right argument ensures then that “Most cats are alive” is true. What makes the monotonicity universal nontrivial is that it is easy to imagine quantifiers that do not satisfy it. Examples of such quantifiers abound among the meanings of complex determiners: “an even/odd number of” or “exactly 2 or 5,” etc. This commonness makes the lack of simple quantifiers not satisfying the monotonicity universal especially puzzling and in need of an explanation. In the following, we will follow the terminology in Barwise and Cooper (1981) and use simply “monotonicity” to refer to monotonicity in the right argument and “persistence” to refer to the equivalent property in the left argument.

The previous work proposed to explain the universal of monotonicity in terms of the greater learnability of monotone quantifiers. Chemla, Buccola, and Dautriche (2019) show that in limited contexts, humans have a bias in learning that favors a weaker version of monotonicity, *connectedness*. Moreover, a post hoc analysis suggests that rules corresponding to monotone quantifiers are easier to learn than other rules (see Chemla, Dautriche, Buccola, & Fagot, 2019, for a similar experiment with baboons). This gives some preliminary evidence that humans find monotone quantifiers easier to learn.

Brochhagen, Franke, and van Rooij (2018) develop an iterated learning model of the evolution of monotonicity, and conclude that monotonicity evolved in a population of pragmatically skillful agents in response to a combination of pressures from learnability and communicative accuracy. The children in Brochhagen et al. (2018) perform Bayesian inference, combining the production data from the previous generation with their own learning biases. The agents are biased for meanings that are easier to describe in a language of thought (LOT), which encodes set‐theoretic relations between A and B. The agents' preference for monotone quantifiers therefore is a direct consequence of the way that the agents' LOT is specified. The result of the model is sensitive to the hand‐coded details of the LOT, calling for independent empirical validation (cf. Carcassi, Schouwstra, & Kirby, 2019, for a Bayesian model of the evolution of monotonicity in the semantics of gradable adjectives). Moreover, this Bayesian model of the evolution of quantificational monotonicity only considers a world with three states, namely *none*, *some*, and *all*.

Pauw and Hilferty (2012) study the evolution of a system of quantifiers in a population of robots. They show that with semantic restrictions, for example, convexity, agents develop meanings for the quantifiers that lead to successful communication. The aims of Pauw and Hilferty (2012) are different from ours. First, they focus on environmental constraints rather than the way cognition influences the meaning of quantifiers. Second, they use agents that do symbolic reasoning rather than neural networks, and focus on communication rather than iterated learning.

Instead of using Bayesian learners with a hand‐specified prior or robotic agents, we expand Steinert‐Threlkeld and Szymanik's (2019) proposal to use neural networks as a model of acquisition of quantifiers. A neural network is a computational device that can learn to approximate functions by observing tuples of inputs and relevant outputs, and progressively minimizing a suitably defined distance between the true output and the network's own prediction. In the case of a quantifier, the input is a structure that encodes the sets relevant to the quantifier's truth and the output encodes whether the structure verifies the quantifier. In practice, given a structure the neural network outputs a probability that can be interpreted as confidence that the structure verifies the quantifier.

**Fig. 1:**
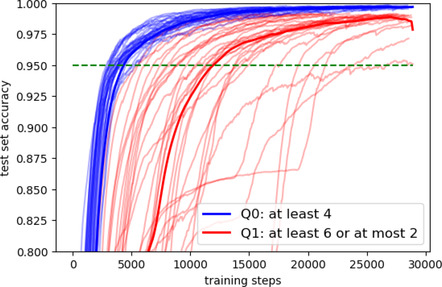
Learning curves on a neural network for the monotone *at least 4* (blue) versus *at least 6 or at most 2* (red). We note here that the model just sees the quantifiers as labels “Q0” and “Q1,” so the linguistic complexity of the expressions is not a factor impacting learning. The x‐axis is the number of training steps; the y‐axis is accuracy (percentage correct) on a test set of examples the network has not yet seen. This was Fig. 4 in Steinert‐Threlkeld and Szymanik (2019). Rather than the feedforward networks of the computational models in this paper, the neural networks in Steinert‐Threlkeld and Szymanik (2019) consist of two stacked long short‐term memory (LSTM) cells (Hochreiter & Schmidhuber, 1997), each with a hidden state of 12 nodes. This allowed for a very general representation of the structure that not only encoded A∩B but also A−B, B−A, and $\bar{A\cup B}$ and a varying number of objects (max 20) in the universe. Thirty networks were trained on each quantifier, and training stopped when the total loss was below 0.01, total mean accuracy for 100 training mini batches was over 99%, or four epochs passed. Adam optimizer was used with a learning rate of $10^{-5}$.

Data about how fast neural networks learn different kinds of quantifiers were produced with the following algorithms. First, two quantifiers are picked such that one satisfies the universal and the other does not. Then, the two quantifiers are taught to a neural network until it has accurately learned them. The crucial information is how long on average it takes neural networks to accurately learn quantifiers that satisfy the universal compared to ones that do not. Various universals were tested in this way. In the case of monotonicity, the data were produced both for a downward monotone and for an upward monotone quantifier. The neural networks were strikingly faster at learning monotone compared to nonmonotone quantifiers. Fig. 1 shows an example.

It should be emphasized that if humans, unlike neural networks, were incapable of learning nonmonotone quantifiers then the approach we take in this paper would be inappropriate. The reason why languages lack simple determiners that do not satisfy the monotonicity universal would simply be that such quantifiers could not be learned at all, and even if introduced would be lost in the following generation. However, there are good reasons for thinking that such quantifiers can be learned. First, as mentioned above participants in Chemla et al. (2019) learned rules corresponding to quantifiers that did not satisfy the monotonicity universal. Second, nonmonotone quantifiers such as “either none or all” are easily understandable, suggesting that while cognitively complex they are well within the capabilities of human cognition.

As discussed above, knowing that meanings with certain features can be learned more easily only goes some of the way in explaining the features' universality across various languages. A full explanation also needs to show that the structure can and eventually will be reached by processes of cultural evolution. In the rest of this paper, we develop an iterated learning model of the cultural evolution of quantifiers that embeds the learning model of neural networks, and show that monotonicity reliably emerges.

## 3 Experiment 1: Cultural evolution of quantifiers

### 3.1 Methods

#### 3.1.1 Iterated learning

Iterated learning models start with two groups of agents, the first and second generations. Some (and possibly all) agents in the first generation—the *cultural parents*—are associated with one or more agents in the second generation—their *cultural children*. A set of linguistic production data are generated by each cultural parent for each of their cultural children. Based on these data, each cultural child tries to approximate its cultural parent's language . Once the agents in the second generation have each learned a language, a third generation is created. In the succeeding steps, the process is repeated with agents in each generation acting as cultural parents and the new agents in the following generation as cultural children. The cultural transmission process is iterated for some number of generations. Each cultural family line is called a *chain* of iterated learning; see Fig. 3 for an illustration of the iterated learning.

Crucially, the agents do not learn their cultural parent's language perfectly. There can be various reasons for this. First, there can be a bottleneck in learning. This happens when the child does not observe everything that is needed to perfectly reconstruct the language, and therefore has to guess some aspects of it. The number of data points given to the children is fixed for all generations and agents and is called the *bottleneck size*. The second reason is that children might not have perfect memory or perfect reasoning abilities, and might therefore learn languages that do not perfectly conform to the given data. In this case, the more rational the child, the closer the learned language will be to the cultural parent's language. However, even given a perfectly rational agent and production data for all world states, it might be impossible to perfectly reconstruct the parent's language. This is because the cultural parents might produce language in a way that is stochastic rather than deterministic. This can make the language harder to approximate and impossible to learn perfectly, constituting a third possible reason for imperfect reconstruction.

The changes introduced by each child accumulate over generations. Often, these changes are not completely at chance, but rather tend to be consistent across agents. Therefore, languages tend to change in the same way in different chains over time. Eventually, the iterated learning chains will mostly move around a part of the language space that can be reliably learned with the amount of data produced by each cultural parent. In sum, iterated learning is a way to study how the cognitive system of the children determine which languages one should expect to see spoken in a population of such agents. The crucial individual level components of an iterated learning model are the set of possible languages, and the way the agents learn them.

In the context of iterated learning models, Bayesian children with a prior that favors languages with a shorter description in a LOT and agents based on neural networks behave differently. After observing only little data, Bayesian children will end up speaking a very regular, simple language, while neural networks will mostly keep their randomly initialized behavior, speaking very unstructured and hard to describe languages. When more data are available, Bayesian agents biased toward simplicity in a LOT will speak the simplest among the languages that explain the observed data, while neural networks will be able to better pick up on patterns in the data. Therefore, while chains of iterated learning will stabilize on simple languages in population of Bayesian agents, in populations of neural children unexpected languages might emerge depending on what patterns the networks can most easily pick up from the data.

In the following, we look at the evolution of the meaning of simple determiners. In these computational models, we do not explicitly formalize the difference between the simple and the nonsimple determiners that can be obtained, for example, by compositional means. The signals in the computational models nonetheless more naturally correspond to real‐world simple signals rather than complex signals. This is because only the meaning of simple signals can naturally be described as being learned, while the meaning of complex signals is inferred from the meaning of the constituent signals. Therefore, the computational models are best interpreted as modeling the evolution of the meaning of simple determiners.

#### 3.1.2 Model of structures, quantifiers, and language

The computational model we propose is concerned with the evolution of the meaning of type ⟨1,1⟩ quantifiers as expressed by simple determiners such as “all” and “several,” or equivalently the evolution of quantified NPs such as “some carrots” or “many people.” In particular, we will not look at type ⟨1⟩ quantifiers as expressed by nonquantified NPs (“Marianne,” “Berta,” “here,” “there,” “this,” “that”).

Since our focus in this paper is on the evolution of the monotonicity universal, we do not need to contrast the monotone quantifiers with the full variety of nonmonotone quantifiers. Rather, we restrict our attention to the subset of quantifiers that are *conservative* and *universe independent*. These, next to the monotonicity universal, are two prominent semantic universals distinguishing natural language quantifiers from all logically possible quantifiers. Universe independence means that extending or shrinking the universe of discourse has no effect on the truth value of the quantifier sentence as long as the left and right arguments are unchanged. For instance, “every” expresses a universe independent quantifier: the truth of “every cat sleeps” is unaffected by variations in the world that do not affect the set of cats and the set of sleeping things. Conservativity means that only the part of B that is common to A matters for the truth value of the sentences. In other words, the elements in B∖A can be safely ignored when determining the truth value. This amounts to saying that for a conservative quantifier Q, evaluating the truth value of Q(A)(B) only requires knowledge of two of the three: A, A−B, A∩B. For instance, “every” expresses a conservative quantifier: the truth of “every dog barks” is unaffected by variations in the set of barking things that are not dogs (For more details on these universals of quantification, see Peters & Westerståhl, 2006).

Given universe independence and conservativity, the truth of any quantifier depends only on which of the elements of A are also elements of B, and which are not. Assuming conservativity and universe independence, both reduces the number of possible quantifiers that agents can speak and simplifies the model of each quantifier, since only A and A∩B need to be encoded. Nonetheless, the set of conservative and universe independent quantifiers contains quantifiers that both conform and do not conform to the monotonicity universal. An example of the latter is the conservative and universe independent complex determiner “Fewer than two or more than four.” The possibility of such quantifiers is crucial in the context of our model, as it means that our restrictions on the set of quantifiers do not by themselves imply the conclusion that quantifiers satisfying the monotonicity universal are widespread. In fact, as we show next only a small proportion of the quantifiers that can be modeled with our representation satisfy the monotonicity universal.

Assuming conservativity/universe independence and a fixed set A with cardinality n, we can represent the part of the world—called a *structure*—that is relevant to determining the truth value of a quantifier as a Boolean vector of a fixed length n. Each element i of the structure represents an object $o_{i}$ in A. Each element has value T (true) iff the object corresponding to that bit is also an element of B, and value F (false) otherwise. For instance, the vector [F,T,T] would model a situation where $A=\{o_{1},o_{2},o_{3}\}$ and $o_{2}$, $o_{3}\in B$. The set of structures is the set of all Boolean vectors with n components, representing the set of possible relations between a fixed A and any possible B. We call $M^{\prime }$ a *substructure* of a structure M iff $M^{\prime }$ is F everywhere where M is F. For instance, [F,T,T,F,F] is a substructure of [F,T,T,T,T]. Note that each structure is a substructure of itself. In intuitive terms, a substructure of M is a situation where some of the objects that were in B in M have been moved outside of B to A∖B.

We represent a *quantifier* as a function from structures into {T,F}, the single Booleans. An example of a quantifier is Q(M)=T iff the structure M is T at two or more indices, and false otherwise, meaning “at least two.” Since for A of size n there are $2^{n}$ different structures, each quantifier is a $2^{n}$‐sized Boolean vector. Each element of the quantifier vector corresponds to a structure and has value T iff the structure verifies the quantifier and F otherwise.^7^

To see how this works in practice, consider a set A of size 3. There are $2^{3}=8$ possible ways in which any other set B can overlap with A. Each of these is modeled as a vector of size 3. For instance, [F,T,T] says that the second and third object of A are also elements of B, but the first is not. The English expression “all As are B” is modeled for the given A by a Boolean vector of size 8 that has value T at the index corresponding to the structure [T,T,T] and F otherwise. If the structures are ordered lexicographically^8^ and the last structure is therefore [T,T,T], then the quantifier corresponds to the vector [F,F,F,F,F,F,F,T]. We call a quantifier *degenerate* iff it corresponds to a vector of identical elements, Fs or Ts. A degenerate quantifier corresponds intuitively to a quantifier that is true (or is false) of every structure.

In sum, each structure is a certain combination of A and A∩B, and is modeled with a Boolean vector of length |A|, where A is fixed throughout the numerical experiment. The Boolean vector is true for at the indices corresponding to elements of A∩B, and false for the elements corresponding to A−B. Each quantifier Q, for a fixed A, is modeled as a Boolean vector where each element corresponds to a structure, and is true at the indices corresponding to Bs that verify Q(A)(B) and false at all other indices.

Each agent encodes a single quantifier within a neural network. Given a structure, an agent produces a truth value using its own neural network. The next two sections detail the connection between the neural networks and the agent's behavior.

#### 3.1.3 Neural networks

Based on the aforementioned learnability results of Steinert‐Threlkeld and Szymanik (2019), the agents that make up the generations in our iterated learning setup are *neural networks*. Each network has n input neurons (one for each bit of a vector corresponding to a structure) and one output neuron (encoding the network's confidence in the truth of the quantifier for that input), with two hidden layers of 16 neurons each and ReLU activation functions except on the last layer, where a sigmoid function is applied to squeeze the output in the (0,1) interval.^9^ Batch normalization is performed in the second to last layer to improve the networks' performance. We used binary cross‐entropy to measure the difference between the parent's output and the child's prediction. We made these design choices so that the networks had enough expressive power to represent many quantifiers, including complex ones. Future work will analyze the effect of architecture choices on the results presented next. The networks and learning, which will be described in the next section, were implemented in PyTorch (http://pytorch.org).

A network in the computational model learns from input/output pairs using a fancier version of gradient descent called Adam (Kingma & Ba, 2015). The network receives a number of true input/output pairs, which it iterates over in small batches. For each batch, it guesses the correct outputs for the inputs, and then updates its parameters (weights and biases connecting the neurons) in such a way that its future outputs are guaranteed to be closer to the truth (for general introductions, see Goodfellow, Bengio, & Courville, 2016; Nielsen, 2015). Because this style of learning is fairly gradual, we introduce one more parameter to our simulations, namely *number of epochs*: this is how many times the network processes its training set in each generation. In other words, the network sees a portion of its cultural parent's language, which we call its *bottleneck size*, but gets to learn from that portion number‐of‐epochs times.^10^ We do not give independent cognitive interpretations to the bottleneck size and the number of epochs, but rather just interpret both as contributing to the total amount of data that the child gets to learn from.

At the moment, it is unclear to what extent the biases of neural networks correspond to those of humans. As we show in Appendix B, the results of the models mentioned below are robust across different optimizers. More in general, the plausibility of backpropagation as a model of learning in the human brain has been explored in recent literature (see Whittington & Bogacz, 2019 for an overview up to 2019). Lillicrap, Santoro, Marris, Akerman, and Hinton (2020), Millidge, Tschantz, Seth, and Buckley (2020), Millidge, Tschantz, and Buckley (2020) proposed approximations of backpropagation that could be implemented in the brain. Moreover, Song, Lukasiewicz, Xu, and Bogacz (2020) recently bridged some technical gaps to showing that the brain might be able to perform backpropagation exactly, rather than just approximately.

#### 3.1.4 Model of the agents

The life of each agent in the computational model goes through two stages. In the first phase, the agent learns a quantifier given data from the previous generation. The data consist of a set of tuples ⟨structure,judgment⟩. The judgment is a single bit expressing whether the quantifier used by the agent's cultural parent is compatible with the structure. These data are used to train the agent's neural network as described in the previous subsection.

In the second stage of their life, after acquiring a language agents produce data used to teach to the following generation. To produce these data, the agent is prompted with randomly chosen structures. Production works as follows. The agent feeds an observed structure to its neural network. The neural network returns a number in the [0,1] interval. Then, the agent rounds the number and returns it. The returned number expresses whether the agent's quantifier is compatible with the structure that the agent observed. The way in which this number should be interpreted is discussed in more detail in Appendix A. The production behavior is deterministic, since an agent always produces the same bit given the same structure.

#### 3.1.5 Measures of monotonicity

According to the standard definition, monotonicity is a binary property. A possible way of analyzing the results would be to find the proportion of monotone languages in every generation. However, some quantifiers are intuitively more monotone than other quantifiers. For instance, consider the three quantifiers “some,” “between 3 and 5,” and “an even number of.” While “some” is monotone and the other two quantifiers are not, intuitively “an even number of” is the least monotone of the three, as it cannot be encoded with a finite Boolean combination of monotone quantifiers. To track finer changes in monotonicity level over time, we define a graded measure of monotonicity.

We measure upward monotonicity in information‐theoretic terms as the proportion of uncertainty in the output of a quantifier that is removed after knowing that there is a substructure where the quantifier is true, that is, a 1.^11^ For a perfectly upward monotone quantifier Q, if a structure M has a substructure to which the quantifier assigns 1 then Q will assign 1 to M. Therefore, for an upward monotone quantifier all the uncertainty is removed and the measure has value 1.

More formally, call M the set of all structures. Let {M,F,P} be a probability space with P a uniform discrete probability function and $F=2^{M}$. Then, define two random variables $1_{Q}$ and $1_{Q}^{≺}$ as follows, with M∈M:

$$\begin{array}{cc} 1_{Q} & =\left\{\begin{array}{cc} 1 & \;\mathrm{if}Q(M)\\ 0 & \;\mathrm{else} \end{array}\right.\\ 1_{Q}^{≺} & =\left\{\begin{array}{cc} 1 & \;\mathrm{if}\exists M^{\prime }.M^{\prime }\mathrm{is}a\mathrm{substructure}\mathrm{of}M\wedge Q\left(M\right)\\ 0 & \;\mathrm{else}. \end{array}\right. \end{array}$$

In words, $1_{Q}$ is 1 if Q verifies a random structure, and 0 otherwise. $1_{Q}^{≺}$ is 1 if a random structure has a substructure that verifies the quantifier. The entropy of $1_{Q}$, $H(1_{Q})$, quantifies the uncertainty about what truth value Q will assign to a structure. The conditional entropy $H(1_{Q}|1_{Q}^{≺})$ quantifies the uncertainty about what Q will assign to a structure, given that one knows whether the structure has a substructure that verifies Q. $H(1_{Q}|1_{Q}^{≺})$ is minimized (attains value 0) for a perfectly upward monotone quantifier: if you know that a structure has a true substructure, and the quantifier is upward monotone, you know the truth value of that structure. The difference between the entropy and the conditional entropy between these variables is known as the mutual information:

$$I\left(1_{Q};1_{Q}^{≺}\right):=H\left(1_{Q}\right)-H\left(1_{Q}|1_{Q}^{≺}\right).$$

This measures how much information $1_{Q}^{≺}$ provides about $1_{Q}$. For a perfectly upward monotone quantifier, $H(1_{Q}|1_{Q}^{≺})=0$, and so $I\left(1_{Q};1_{Q}^{≺}\right)=H\left(1_{Q}\right)$. In other words, for an upward monotone quantifier, knowing which structures have a true substructure provides as much information as knowing the entire quantifier.

While this roughly captures what we want from a measure of upward monotonicity, it needs to be normalized to form a degree that applies across quantifiers, since $0\leq I\left(1_{Q};1_{Q}^{≺}\right)\leq H\left(1_{Q}\right)$. We do this by dividing by $H(1_{Q})$, moving the upper bound to 1. Overall then, we measure upward monotonicity as

$$\begin{array}{cc} \mathrm{mon}(Q) & :=\frac{I(1_{Q};1_{Q}^{≺})}{H(1_{Q})}\\ & =\frac{H\left(1_{Q}\right)-H\left(1_{Q}|1_{Q}^{≺}\right)}{H(1_{Q})}\\ & =1-\frac{H(1_{Q}|1_{Q}^{≺})}{H(1_{Q})}. \end{array}$$

To see how this measure tracks intuitions, consider the previously mentioned quantifiers “some,” “between 3 and 5,” and “an even number of.” “Some” gets monotonicity 1.0 because knowing whether a structure has a substructure that verifies “some” eliminates all uncertainty about the truth of the structure. An agent whose quantifier is “between 3 and 5” has degree 0.7517 and one with “an even number of” has degree 0.001, which captures the intuitive order of monotonicity of these quantifiers.

Up until this point, we have focused on a measure of upward monotonicity. The measure above can be straightforwardly modified to measure downward monotonicity, by replacing the variable $1_{Q}^{≺}$ for the variable $1_{Q}^{≻}$, which is true when a structure has a true superstructure. We discuss the way we calculated overall monotonicity, including both downward and upward, in more detail in Appendix A.

**Fig. 2:**
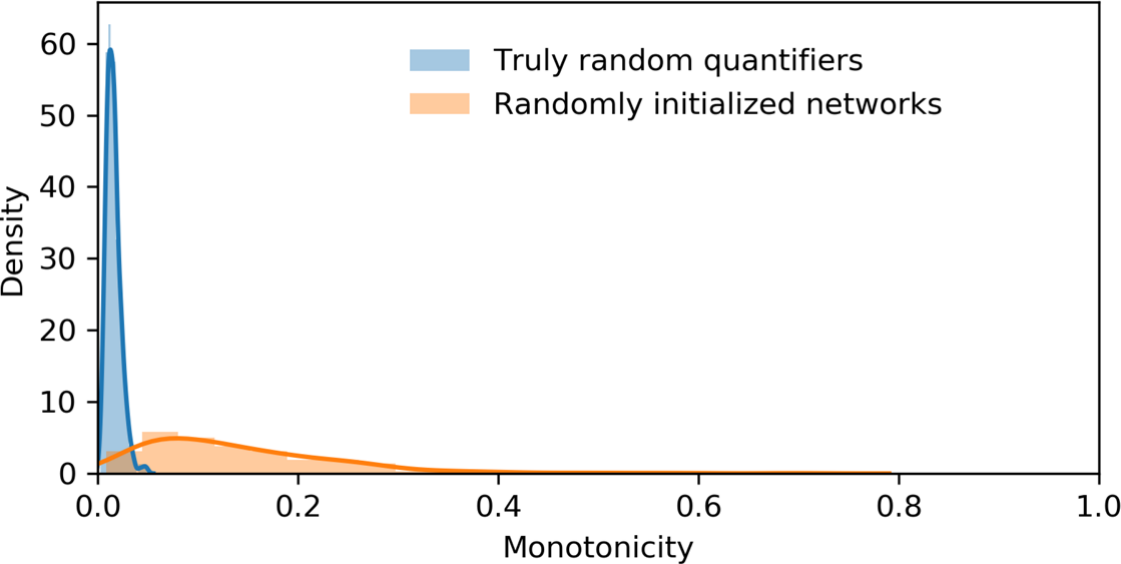
Kernel density estimation of the distribution of degrees of monotonicity from a sample of 300 completely random quantifiers and 300 random neural network agents (max structure size of 10). The *x*‐axis is the measure of monotonicity we describe in the main text.

We compare the results of the simulation to the distribution of the measure in randomly generated quantifiers. There are two different random distributions of quantifiers. On the one hand, there are the quantifiers instantiated by randomly initialized agents. On the other hand, there are the quantifiers sampled uniformly from the space of possible quantifiers. These two distributions are depicted in Fig. 2. While the completely random quantifiers have a narrower distribution, both types of random distribution are very skewed toward low degree of monotonicity. This makes sense: monotonicity is a relatively rare property, and so should not be expected to appear randomly. We now turn to the results, showing that higher degrees do emerge via iterated learning.

#### 3.1.6 Materials

**Fig. 3:**
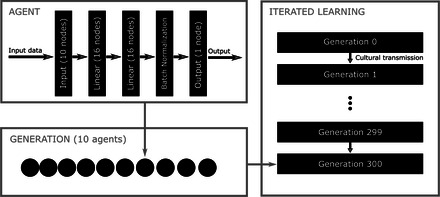
Overview of the computational model. Each run of the iterated learning algorithm consists of generations (right subplot). Languages in generation 0 are initialized at random. Each generation consists of agents (bottom left plot). Each agent in each generation (after the first) is associated with a randomly chosen agent from the previous generation. The latter (called the *cultural parent*) produces linguistic data on which the former (called the *cultural child*) is trained. Specifically, the language of each agent is encoded in a neural network (top right plot). The network of the cultural child is trained on tuples of structures and truth‐value judgments produced by the neural network of the cultural parent. Each agent in the model encodes one quantifier.

For our experiments, we used a fixed structure size of 10 (which, recall, is also the size of the input to the agents), with 10 agents in each generation, and varied the bottleneck size (200, 512, 715, 1024) and the number of epochs (4 and 8). For each setting of those two parameters, we ran 20 trials. The code, data, and instructions for running experiments may be found at https://github.com/thelogicalgrammar/NeuralNetIteratedQuantifiers. Fig. 3 shows an overview of the computational model.

### 3.2 Results

**Fig. 4:**
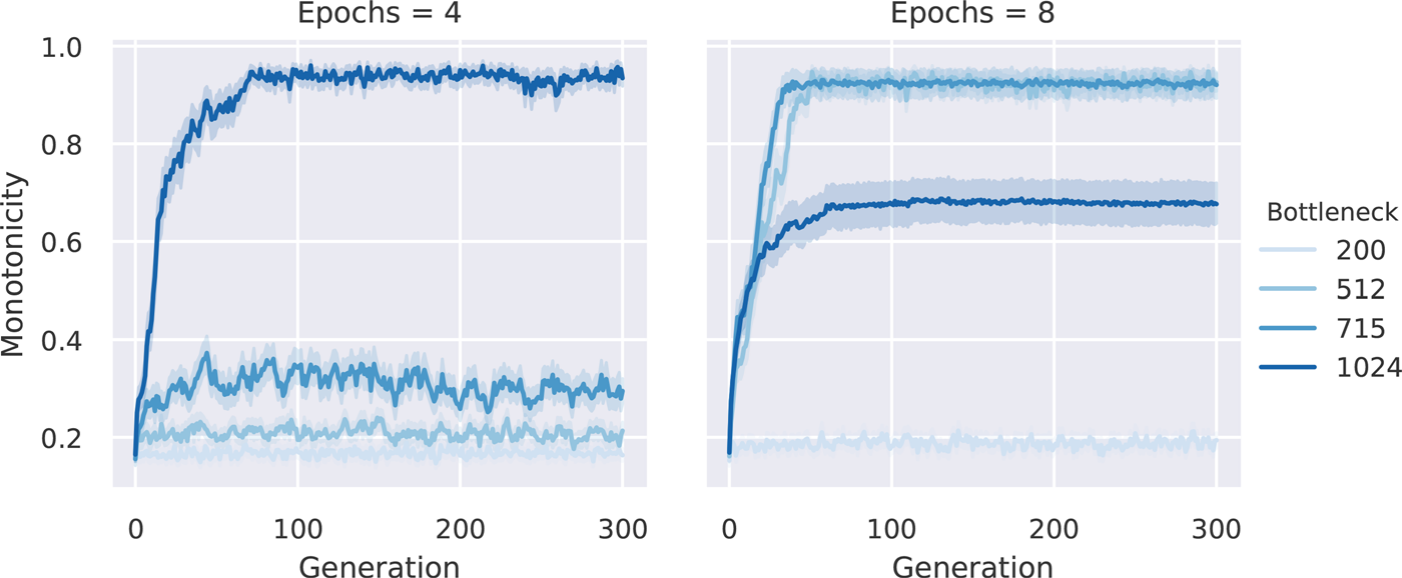
The simulation was run 20 times for each combination of bottleneck size and number of epochs in a population of 10 agents and a maximum structure size of 10. The plot shows how the average monotonicity level across all languages changes over 301 generations (shaded area show the 95% confidence interval). Convergence to monotonicity depends on how much the children's neural networks are trained, which itself depends on the number of epochs and the bottleneck size. With small bottleneck and few epochs, monotonicity does not evolve. With a bigger bottleneck size and more training epochs, monotone languages become widespread. However, increasing the training data further tends to impede the development of monotone languages.

The first result is that monotone quantifiers evolve consistently and rapidly for some values of the simulation parameters. More specifically, the evolution of monotonicity depends on the bottleneck size and the number of epochs, that is, how much of the cultural parent's language is observed by the cultural child; see Fig. 4 for the results. If the networks get too much input, they learn the quantifier accurately and change is very slow. If the networks get too little input, the learning has little effect and no pattern emerges. If languages are somewhat stable across generations, but enough variation is allowed by not overtraining the cultural children, monotonicity evolves.

A second result is that the monotone quantifiers that emerge are in large part nondegenerate. With Bayesian agents that have a bias for simplicity, degenerate languages become widespread under pure iterated learning (Kirby, Tamariz, Cornish, & Smith, 2015). Here, however, degenerate quantifiers only constitute a small minority of the evolved languages (about 0.005% of all quantifiers). This result is consistent with the fact that neural networks produce noisy output unless they are trained on a pattern, while Bayesian learners biased toward simplicity in a LOT learn the simplest among the languages compatible with their observations. As we show in Section 4, degenerate languages can be learned by neural networks, but are less stable than other patterns.^12^

The third result of the simulation is that most nondegenerate monotone quantifiers fall in one of a few types. About 79% of the perfectly monotone quantifiers show the following pattern: there is some index i such that the quantifier—call it $Q_{i}$—assigns 1 to a structure iff the structure is 1 at i (or an equivalent pattern obtained by switching 0 and 1 uniformly in the structures and/or in the quantifier). $Q_{i}$ is true iff $o_{i}$, the object represented by index i, belongs to the set B.^13^. Therefore $Q_{i}\left(A\right)$ functions much like a proper noun for $o_{i}$. Just like “Anna is human” is true iff Anna belongs to the set of humans, “$Q_{i}\left(A\right)$ is B” is true iff $o_{i}$ belongs to the set B.

For other monotone quantifiers $Q_{\left\{j,k\right\}}$, there are two indices j,k (with j≠k) such that $Q_{\left\{j,k\right\}}$ assigns 1 to a structure iff the structure has value 1 at both j and k (or, again, an equivalent patterns obtained by switching 0 and 1 in the structures and/or in the quantifier). $Q_{\left\{j,k\right\}}$ is true iff B contains two specific elements of A, and false otherwise.^14^ It functions like the conjunction of two proper nouns. Like “Anna and Rob are human” is true iff Anna is human and Rob is human, “$Q_{\left\{j,k\right\}}\left(A\right)$ is B” is true iff $o_{j}$ is B and $o_{k}$ is B.

## 4 Experiment 2: Shuffling the individuals

The majority of the languages that evolved in Experiment 1 are ultrafilters, which can be naturally interpreted as naming one of the objects in the structure. The neural networks can exploit a particularly simple strategy in the acquisition of ultrafilters, which consists in copying or reversing the input at the index of the relevant object. For instance, if the quantifier is naming the third object in the structure, the neural network simply needs to output 1 when the third component of the input vector is 1, and 0 otherwise. The agents are capable of learning this simple rule with little input data, making the ultrafilters very stable in the process of cultural evolution.

While the quantifiers that evolve in the first experiment are indeed monotonic, they are unlike quantifiers in natural language. Specifically, the truth of the corresponding natural language quantifiers would depend on a specific object, and therefore they do not satisfy another important universal of quantification, *quantity*.^15^ To further test whether neural networks have a preference for monotone quantifiers, we introduce a small change in the model of learning that prevents the networks from exploiting the simple strategy of copying the input at an index. We find that monotonicity evolves even when quantifiers cannot encode information about the identity of specific individuals.

### 4.1 Methods

#### 4.1.1 Model of transmission

**Fig. 5:**
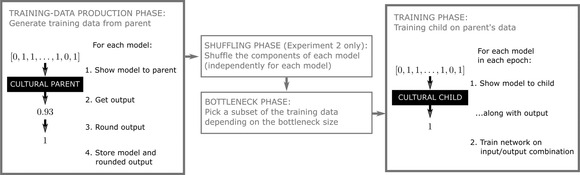
Schematic representation of how cultural transmission from a cultural parent to a cultural child happens in the model. Note that the only difference between Experiments 1 and 2 is in the shuffling of the models before the cultural child is trained.

In Experiment 1, the child observes tuples consisting of a structure and the output of the parent's neural network for that structure. In Experiment 2, the parent observes a structure and produces an output in the same way as in Experiment 1. However, in Experiment 2 the child observes, along with the parent's original output, a shuffled version of the input observed by the parent. In the shuffled structure, the total number of 1s and 0s is the same as in the original structure observed by the parent, but their order can change; see Fig. 5 for a visual explanation of the difference between Experiments 1 and 2.

The shuffling introduced in Experiment 2 requires a change in the interpretation of the structures. In Experiment 1, each index of a structure represents a specific object in A, which is stable across observations by individual children as well as across generations. On other hand, in Experiment 2 the parent and the child observe structures that are different (up to permutation) within a single instance of language production. Shuffling can then be interpreted simply as a way to prevent the parent from referring to individual elements of A across productions, and thus to prevent the evolution of ultrafilters. However, shuffling can also be interpreted more substantially as reflecting the fact that real language users often do not keep track of the specific individuals belonging to a set. For instance, quantifying over the set of glasses—“Q glasses are clean”—does not require language users to keep track of the identity of individual glasses over time, or even within a single instance of communication.

It is worth noting that while in Experiment 1 the data observed by children are consistent, that is, a structure is always associated with the same output, the data observed by the child might not be consistent in Experiment 2, despite parents producing consistent data. Because the input is shuffled independently for each observation, two different structures with the same number of 1s that receive different outputs in the parent's language might be shuffled onto identical structures before the child observes them.

#### 4.1.2 Measure of quantity

In Experiment 2, the quantifier cannot convey information about specific objects. In other words, a quantifier can be reliably preserved across generations only if the values it attributes to a structure is invariant across all permutations of the structure. The only quantifiers that can in principle be transmitted perfectly are therefore *permutation invariant*. Like the monotonicity universal, permutation invariance has also been proposed for a universal of quantificational semantics (Keenan & Stavi, 1986). (For a more extensive discussion of the quantity universal, including a stronger formulation in terms of isomorphism, see Peters & Westerståhl, 2006.) This constraint rules out quantifiers, like the “first three,” as candidate meanings for natural language determiners. A quantitative quantifier attributes the same truth value to any two structures that can be permuted into each other. The only information that all permutations of a given structure share is the number of 0s and 1s. Therefore, all that matters in determining the output of a neural network encoding a quantitative quantifier is the number of 1s in the structure, or equivalently the size of A∩B, and |A|, which is known and fixed throughout the experiment.

Children can fail to acquire a quantitative quantifier for two reasons. First, a child may pick up on noise in the observed data and memorize spurious observed associations between the shuffled structure and their parent's output. Second, the child might not change its original output for some of the structures, regardless of whether it is consistent with the observed data. Approximately quantitative quantifiers can evolve in the iterated learning despite noise if agents learn that the truth of the quantifier depends on the number of 1s in the structure.

While permutation invariance as defined above is a binary property, in order to quantify the extent to which the iterated learning chains stabilize on quantitative quantifiers, we develop a graded measure of quantity for quantifiers. This measure analyzes the degree of quantity of a quantifier Q as the proportion of information about the truth of a structure for Q that is eliminated by knowing the number of 1s in the structure. Formally, we define a random variable #, which is the number of 1s in a structure (with the same probability space as in Section 3.1.5):

#=NumberofTsinM.

Then, the degree of quantity for a quantifier Q is one minus the (normalized) conditional entropy of $1_{Q}$ given #:

$$\mathrm{qua}\left(Q\right):=1-\frac{H(1_{Q}|\#)}{H(1_{Q})}.$$

In the structures corresponding to fully quantitative quantifiers, such as “between 2 and 4,” knowing the size of A∩B leaves no uncertainty about whether the structure is true or false for the quantifier (since the size of A is fixed). Therefore, the conditional entropy of the truth value of a structure given the size of A∩B will be 0, and the quantifier's degree of quantity 1.

### 4.2 Results

**Fig. 6:**
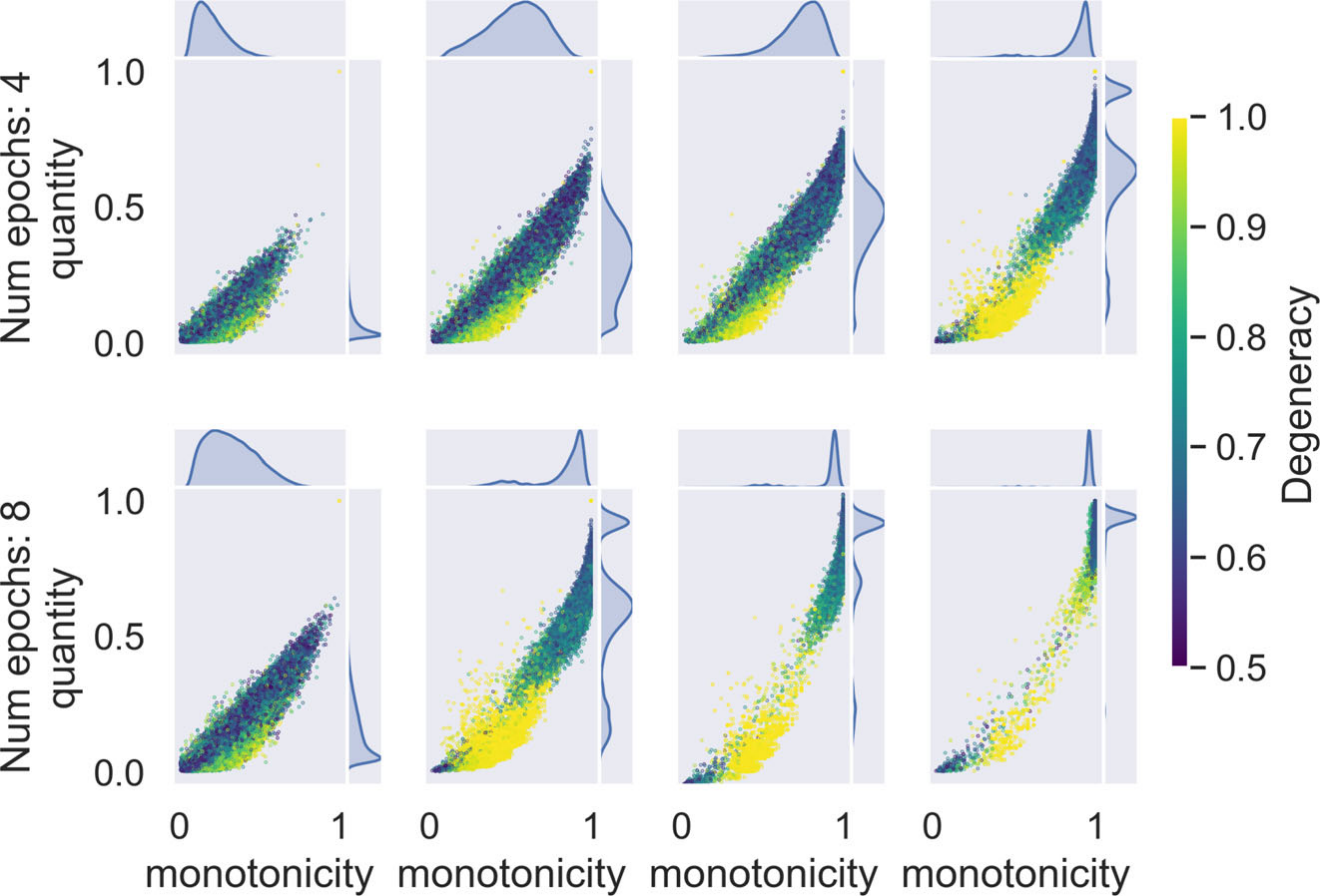
The simulation was run 20 times for each combination of bottleneck size and number of epochs in a population of 10 agents and a maximum structure size of 10. Each dot shows the quantity and monotonicity level of the language spoken by one agent in one generations, color‐coded by the similarity to a degenerate quantifier. All agents in all generations are shown. When agents observed enough data to preserve some structure from their parents' language, the iterated learning chains soon converge to either nearly degenerate quantifiers (yellow dots) or vague proportional‐like quantifiers (high degree of monotonicity and quantity), observable in the plot as two clusters of languages. When more data are available, the evolved languages approximate more closely the two types of quantifier.

We ran Experiment 2 with the same combinations of parameters as Experiment 1. Results can be seen in Fig. 6. The main result of Experiment 2 is that, despite the shuffling, the evolved quantifiers have in large part a high degree of monotonicity. This confirms the results of Experiment 1, showing that the evolution of monotonicity is a robust feature of culturally evolved quantifiers.

**Fig. 7:**
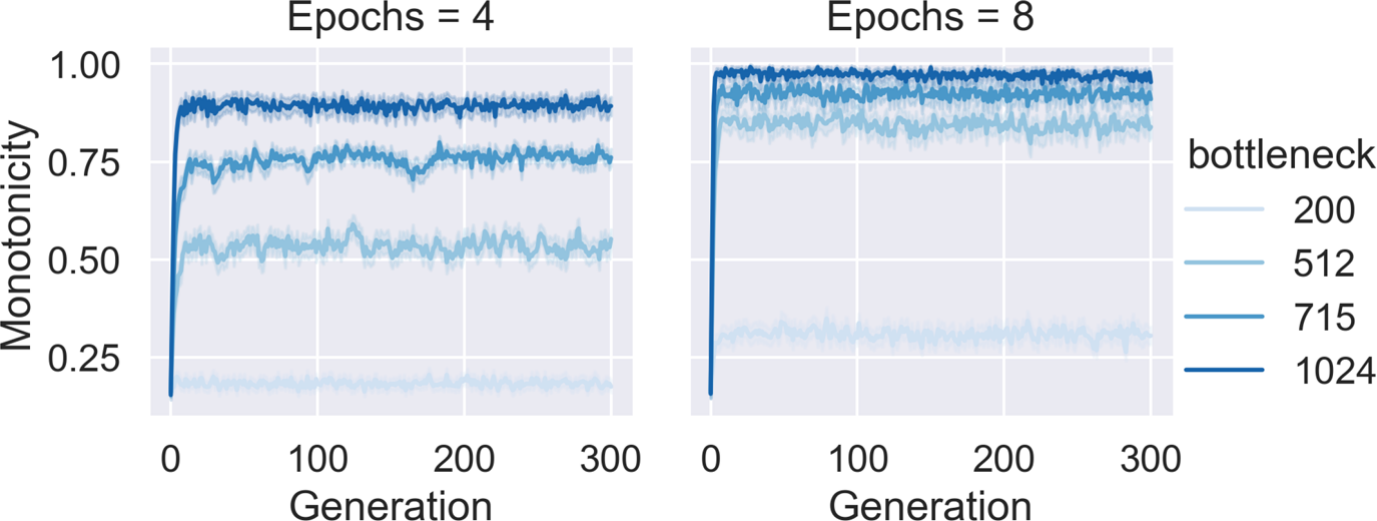
Monotonicity of languages evolved in Experiment 2 by generation. When agents observe more data, they tend to stabilize on higher levels of monotonicity. In combination with Fig. 6, which shows that degenerate quantifiers concentrate at low levels of monotonicity, this shows that in chains where agents see more observations, degenerate languages concentrate in early generations.

While the shuffling puts a strong pressure toward the evolution of quantitative quantifiers in the model, it does not in itself induce the evolution of monotone quantifiers. To see why, note that structures in the computational model can encode perfectly quantitative quantifiers—for example, “between two and four”—which are not monotonic. More specifically, for structures of size n in the computational model, there are ∑i=0n+1n+1i=2n+1 perfectly quantitative quantifiers, and of these only 2n+2 are monotonic. For the structures with 10 components in the simulations above, only 1.07% of quantitative quantifiers are also monotonic. The small proportion of monotone out of the quantitative quantifiers shows that the evolution of monotonicity cannot be explained as a side effect of the increased degree of quantity (Fig. 7).

The second result of Experiment 2 is that the iterated learning chains consistently stabilize on the same two types of quantifiers, namely nearly degenerate quantifiers and quantifiers with a vague threshold. The first type of quantifiers that evolve, degenerate, or nearly degenerate quantifiers, output the same value for all structures except for a few exceptions. These exceptions, to the best of our knowledge, do not follow from a general pattern but are rather memorized by the networks. We measure degeneracy as the maximum among the proportion of 0s and the proportion of 1s in the quantifier's output. Nearly degenerate quantifiers have a comparatively low level of monotonicity, which can be visualized Fig. 6. This is because the measure of monotonicity is very sensitive to random noise in an otherwise degenerate quantifier. Despite the low level of measured monotonicity, the underlying pattern of degeneracy that the neural networks approximate is a monotone pattern.

**Fig. 8:**
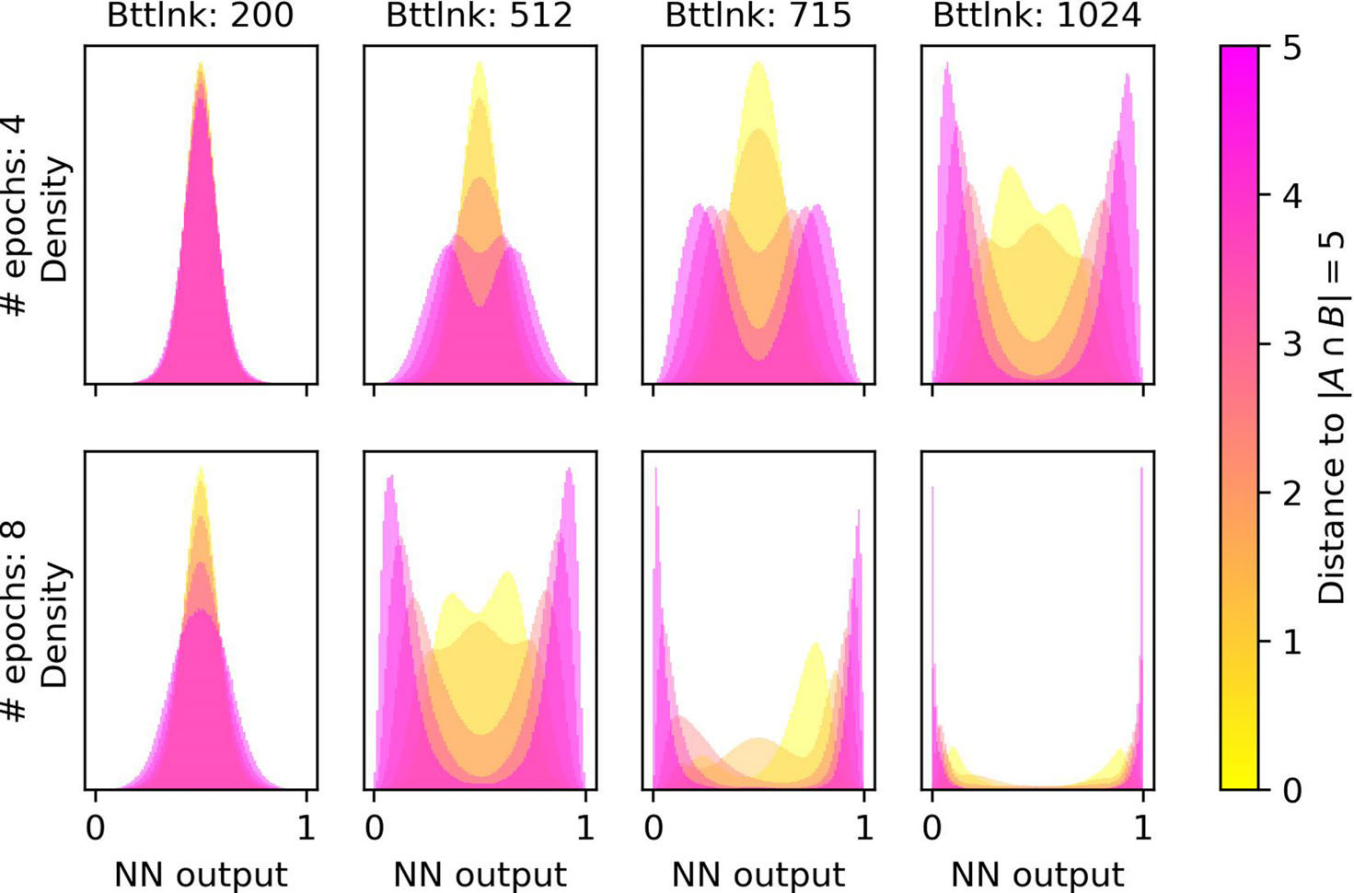
Unrounded neural network output, representing the confidence level in the truth of the quantifier, as a function of the size of A∩B for various parameter regimes. Each distribution in each plot is a kernel density estimate of the output of the neural net for all structures with a given |A∩B|. For visual clarity, the color does not represent |A∩B| directly, but rather encodes |5−|A∩B||, intuitively how the size of |A∩B| differs from the mean size. Nearly degenerate quantifiers have been excluded. As the agents observe more data, they become more confident in how the size of A∩B affect whether the quantifier is true or false. This means intuitively that the quantifier encoded by the agents becomes more crisp.

The second type of quantifier on which the chains of iterated learning stabilize tends to switch output around a specific values of |A∩B|, for example, by outputting 0 for all structures with less than 4 ones and 1 for all structures with more than 4 ones. Thresholds are not crisp, but rather vague: not all structures with the same number of 1s are mapped onto the same output, but the mean output of the quantifier is a monotone function of the number of 1s in the structure. Because of the assumption encoded in the structure of a fixed size for A, it is not possible to determine whether the threshold quantifiers learned by the neural networks depend on the absolute number or on the proportion of 1s in the structure, corresponding, respectively, to cardinal quantifiers and proportional quantifiers from a linguistic point of view.^16^ Further work is needed to disambiguate between these two readings, and we return to this problem in the discussion below. Bracketing the difficulty of drawing the distinction, we call these quantifiers *proportional‐like*. As can be seen in Fig. 8, the amount of vagueness decreases as the agents are trained on more data. With a small bottleneck of 200 observations (left column of plots), agents are uncertain about the output for all structures, while with 8 epochs and 1,024 observations (bottom right plot) the size of A∩B completely determines the output of the quantifier.

The thresholds of the proportional‐like quantifiers that evolve in the experiment are not distributed uniformly across the possible sizes of A∩B. Rather, sizes between 2 and 8 are more common, and quantifiers with transitions at the extreme sizes, that is, 0 and 10, never evolved. A possible reason why proportional‐like quantifiers tend to have nonextreme thresholds is that the pattern of proportional quantifiers with extreme or close‐to‐extreme thresholds is based on the judgment of only a few structures, in the extreme case either the all‐1 structure or the all‐0 structure. The accuracy of transmission is lower for quantifiers whose pattern depends on fewer structures, both because the probability of the child observing and correctly memorizing the crucial structures decreases and because if a pattern is encoded by few structures, it becomes more difficult to distinguish the outputs that are determined by the pattern from outputs that the agent has simply memorized for specific structures, rather than inferred from a pattern.

The result that nonextreme thresholds might have an advantage in cultural evolution complements the previous literature which attempted to explain the evolution of extreme thresholds in the semantics of scalar terms. For instance, Lassiter and Goodman (2017) and Qing and Franke (2014) argue that the emergence of extreme thresholds in natural language is a consequence of a pressure for communicatively accurate signals. The results show that a pressure from learning might counterbalance this pressure from communication, favoring proportional‐like quantifiers with nonextreme thresholds. In the context of a pressure from learning alone, Carcassi, Schouwstra, and Kirby (2020) show that for the simpler semantics of gradable adjectives, learning alone might favor extreme thresholds. We leave the effects of a joint pressure from learning and communication to future research.

Overall, excluding extreme thresholds for the reasons discussed above, all and only the quantitative monotone quantifiers emerged in Experiment 2. While the evolved quantifiers approximate degenerate and proportional quantifiers with noise, meaning that some structures get a truth value that is inconsistent with the quantifier's overall pattern, Fig. 6 shows that as the agents are trained on more data they stabilize on less noisy approximations of degenerate and vague proportional quantifiers. This result shows that, at least among the quantitative quantifiers, neural networks have a bias in favor of monotonicity specifically, rather than strategies which correlate with monotonicity in some but not all cases.

## 5 Discussion

The results from the two experiments presented in this paper have shown that monotonic, nondegenerate quantifiers reliably evolve in a population of neural networks under the sole pressure from learning, showing that learning biases might suffice to explain the universal of monotonicity. This result seems to contradict the previous work in iterated learning showing that, under a pressure from learning alone, languages tend to become degenerate, and motivates experimental research on which quantifiers would be accurately conveyed by human participants (Kirby et al., 2008, 2015). Nonetheless, when the agents in Experiment 2 observe enough data, degenerate quantifiers are transmitted faithfully across generations and are stable in cultural evolution.

The computational model presented in this paper can be straightforwardly extended in various ways. The agents judged their quantifier compatible with a given structure simply by rounding the output of their neural network. An alternative to this is for the agents to accept a structure with a probability proportional to the network's output. Such so‐called *sampling* agents do not straightforwardly instantiate a quantifier, since they can produce inconsistent output when repeatedly prompted with the same structure.

As discussed in the previous section, the threshold quantifiers that emerged in Experiment 2 underdetermine the natural language difference between cardinal and proportional quantifiers. In order to disambiguate between these two possibilities, a more complex representation of structures could be used which allows vectors of different sizes. Cardinality quantifiers would then be the ones whose truth value depends on the number of ones in a structure, while proportional quantifiers the ones whose truth value depends on the proportion of ones. While the feedforward neural networks we used above do not accept variable size input, recurrent neural networks (such as long short‐term memory (LSTM) networks, see, e.g., Hochreiter & Schmidhuber, 1997) could be used which accept sequential data of possibly varying length. Even though this seems like a natural follow‐up linguistic question, we do not pursue it in this paper as it is not directly related to our leading problem, whether monotonicity emerges as an effect of the ease of learnability.

Generalizing the structures and using LSTMs would also allow agents to learn quantifiers that fail to have other proposed universals of quantification, such as conservativity and universe independence. Based on Steinert‐Threlkeld and Szymanik (2019), the evolution of these further universals could be explored in an iterated learning context. This is of particular interest because, while in the above we have looked at quantity and monotonicity separately, these two universals—along with a variety of others—in fact coevolve. Future work can look at the coevolution of these various universals together rather than in isolation.

Beyond the universals of quantification, neural networks have been shown to more easily learn meanings satisfying other semantic universals, such as *veridical uniformity* (Steinert‐Threlkeld, 2019).^17^ An iterated learning extension of such models could also offer insights in the way individual biases are reflected on a population of neural network agents.

Another pressure that might contribute to shape the meaning of quantifiers comes from communication (Kirby et al., 2015). Our results show that learning pressures may be the force responsible for the universal of monotonicity. However, communication might also play a role in this explanation. In particular, the evolution of the universal of monotonicity might be a case where communication and learning push toward similarly structured categories, namely those satisfying the universal of monotonicity. In the case of categories expressed by nouns, the universal property of convexity has been argued to be a consequence of a pressure both from learning (Gärdenfors, 2004) and from communication (Jäger & van Rooij, 2007). As pointed out in Chemla et al. (2019) and Carcassi (2020), the property of convexity is structurally similar to the property of monotonicity as defined above (see cited papers for a more discussion of why this is the case), suggesting that they might be explained by similar pressures.

Beyond monotonicity, while some semantic universals of quantification might have an advantage in cultural evolution because they conform well with learning biases, other universals might evolve because they lead to more successful communication. Therefore, combining iterated learning with a pressure for accurate communication, implemented for instance by direct selection of communicatively successful languages, might help more natural quantifiers emerge. Recent work has looked at the emergence of compositional languages from interacting neural networks (Choi, Lazaridou, & de Freitas, 2018; Foerster, Assael, de Freitas, & Whiteson, 2016; Yuan et al., 2020), but the role of direct selection of languages by communicative accuracy in an iterated learning model with neural agents has not yet been studied (although see Ren et al., 2020, for an implementation of a pressure for expressivity in iterated learning with neural networks). We leave all these exciting possibilities to future work.

The results we presented support the learnability account of the origins of semantic universals of quantification. While the previous work compared quantifiers satisfying semantic universals to quantifiers that do not, we have presented a computational model where the former are selected out of all the possible quantifiers by a process of cultural evolution. Moreover, the preference for monotone quantifiers is not a consequence of an explicitly coded bias for simplicity, but rather of an independently motivated, biologically plausible model of learning. The results therefore suggest that not only are monotone quantifiers easier to learn, but they are also widespread in language *because* of their learnability.

### Open Research Badges

This article has earned Open Data and Open Materials badges. Data and materials are available at https://osf.io/ume39/ and https://github.com/thelogicalgrammar/NeuralNetIteratedQuantifiers.

## Figures and Tables

**Fig. B1:**
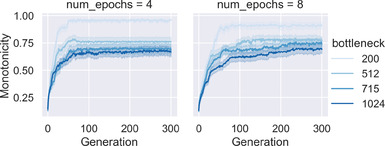
Level of monotonicity by combination of parameters in the first experiment with SGD optimizer. Like for the previous experiments, the main result is observed. Namely, the population develops higher levels of monotonicity than would be expected in a random sample of quantifiers.

**Fig. B2:**
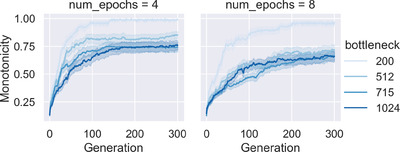
Level of monotonicity by combination of parameters in the first model with SGD + momentum optimizer. The population again develops higher levels of monotonicity than would be expected in a random sample of quantifiers.

**Fig. B3:**
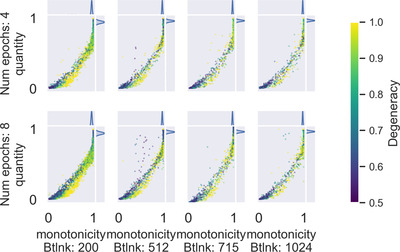
Monotonicity against quantity for each language spoken across all generations in the replication of Experiment 2 above with SGD as optimizer. Like in the results above, the languages cluster in two groups.

**Fig. B4:**
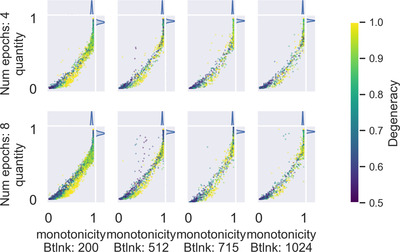
Monotonicity against quantity for each language spoken across all generations in the replication of Experiment 2 with SGD + momentum as optimizer. Like in the results above, the languages cluster in two groups, albeit with a higher proportion of degenerate quantifiers.

## Notes

1. See for instance Hunter and Lidz (2012) for the universal of conservativity in quantifiers. However, see Spenader and de Villiers (2019) for a failed replication, and Zuber and Keenan (2019) for a slightly weakened formulation of the conservativity universal that better conforms to the linguistic data.

2. Note that with “cultural parent,” we do not mean simply the legal parent of a child, but rather any individual from whom the child acquires cultural information.

3. Exactly how to draw the distinction between simple and complex and whether, for instance, *most* is simple or complex, do not matter for present purposes.

4. Informally, a quantifier Q satisfies the universal of quantity if and only if (iff) for any two sets A and B the truth value of Q(A)(B) only depends on the cardinalities of A−B, B−A, A∩B, and $\bar{A\cup B}$. More detail is given in Section 4. For a formal definition of quantity, see Peters and Westerståhl (2006).

5. For more information on generalized quantifier theory from linguistic, computational, and cognitive perspectives, see also Peters and Westerståhl (2006) and Szymanik (2016). Keenan and Paperno (2012) and Paperno and Keenan (2017) present typologically rich data obtained through a questionnaire, but did not ask directly about monotonicity. Bach et al. (1995) also provide a wealth of cross‐linguistic data on quantification in general but not on monotonicity. Matthewson (2008) also reports cross‐linguistic data on quantification.

6. The formulation in terms of determiners' right argument, rather than NPs, comes from Peters and Westerståhl (2006, p. 172). The definition in Barwise and Cooper (1981) technically concerns NPs and not only quantified NPs. In the following, we will focus only on the latter, excluding expressions such as proper nouns, deictic pronouns (“I,” “you”), and demonstrative pronouns (“this,” “that”). We an anonymous reviewer for pointing to this distinction.

7. In practice, while we are interested in type ⟨1,1⟩ quantifiers, the quantifiers in the model are effectively type ⟨1⟩. The type ⟨1⟩ quantifiers that we use in the model are relativizations of the type ⟨1,1⟩ quantifiers (see Peters & Westerståhl, 2006).

8. In this case, lexicographic order is the dictionary order over sequences of letters from the alphabet {F,T} with F preceding T in the order.

9. Any specific number of nodes and input layers has to be picked arbitrarily. We selected an architecture that is quite expressive and capable of learning nonmonotone quantifiers, while still remaining simple. Recent work by Baldi and Vershynin (2019) explores the problem of the capacity of feedforward neural networks. While their results concern networks with a Heaviside activation function rather than ReLU, they conjecture that structurally identical networks with the latter have higher capacity than the former.

10. In some experimental literature—for example, Carr, Smith, Culbertson, and Kirby (2019)—this is also referred to as *exposures*.

11. In the rest of this section, we assume that 1 is to be interpreted as T and 0 as F. For more details, see Appendix A.

12. We thank an anonymous reviewer for pointing out connections between neural networks and Bayesian learners, specifically in connection to previous results on iterated learning. While Griffiths and Kalish (2007) showed that the distribution of languages in iterated learning models will converge to the prior for sampling Bayesian agents, the neural networks' output in our computational model is deterministic, implying that they should be characterized as MAP agents. The convergence behavior of iterated learning is more complex with MAP agents, with the distribution of languages approximately centered around a prior mode and a variance that depends on the specific parameters of the simulation (Kirby, Dowman, & Griffiths, 2007). Moreover, the situation in the current paper is further complicated by the fact that different weight initialization cause different neural networks to infer different probabilities for new data given the same training data, so that they would correspond with Bayesian learners with different prior. As shown by Navarro, Perfors, Kary, Brown, and Donkin (2018), the stationary distribution in a population with heterogeneous biases does not in general have a simple relation to the prior, so that even while knowing the bias of the single agents the results of cultural evolution are not easily predictable. In sum, there are reasons to be skeptical that the results in this paper could easily be reduced to an analytic result in the literature. Despite this, the results of iterated learning are clearly influenced by the agents' biases. An important question is therefore whether the implicit bias of neural networks for monotonicity should be interpreted as a human‐specific cognitive bias or as a feature of any universal learning algorithm. While we do not take a position in this discussion, this paper contributes to the previous literature showing that a range of different learning algorithms have a bias for monotonicity, therefore suggesting the conclusion that any general model of learning will be biased toward monotone categories; see, for example, van de Pol, Steinert‐Threlkeld, and Szymanik (2019) for monotonicity and approximate Kolmogorov complexity and Szymanik (2016) for monotonicity and logic, automata theory, and computational complexity.

13. In set‐theoretic terms, $Q_{i}$ is a *principal ultrafilter* If U is a finite nonempty set, a set F is a principal ultrafilter on U if there is an a∈U such that F={B∈P(U)|a∈B}. $Q_{i}$ is (the characteristic function of) a principal ultrafilter on B because it contains every subset of B that contains i.

14. These are called in set‐theoretic terms *principal filters*. They are not principal ultrafilters because their truth depends on more than one element.

15. We are interpreting the evolved quantifiers as quantified NPs with a fixed A. However, if the quantifiers are interpreted more generally as any type ⟨1⟩ quantifiers, the ultrafilters correspond to proper nouns. The model itself is underdetermined with respect to these two interpretations.

16. Cf. some natural language quantifiers, such as “many” and “few,” which have been argued to be in fact ambiguous between a cardinal and a proportional reading (Partee, 1988).

17. The universal of veridical uniformity can be roughly stated as follows: every responsive verb (i.e., verbs that can take both declarative and interrogative complements) is veridical with respect to declarative complements iff it is veridical with respect to interrogative complements. For instance, “know” is veridical: “Mary knows that James won the race” implies that James won the race, and “Mary knows who won the race” implies that Mary knows that John won the race (assuming that John indeed won the race). For a more rigorous explanation, see Steinert‐Threlkeld (2019).

## APPENDIX A Neural networks and truth values

While the neural networks take structures as inputs, they cannot work with Boolean vectors directly, but rather require bit vectors. Therefore, true and false have to be represented as bits. In the discussion of the interpretation of neural network's inputs and outputs, we have assumed the usual interpretation of 0 and 1 as true and false, respectively. However, this was a simplification. While it is conventional to define 1 as true and 0 as false, this will not work in the context of languages evolved by neural networks, since the networks do not necessarily associate 1 with truth and 0 with falsehood. Moreover, the way bit values are mapped onto Boolean values need not be identical for a network's input and its output.

More specifically, when prompted with a bit string agents produce a single bit. The former models a state of the world, the latter models the compatibility of the agent's quantifier with the world state. While 1 and 0 are often used to represent true and false, as mentioned in the previous paragraph nothing in the simulation implies that neural networks are interpreting 1 and 0 as true and false, respectively, in their input and output. Therefore, if quantifiers attribute a truth value to each structure, the output of an agent underdetermines which quantifier the agent speaks, even when the output for all structures is known. For instance, an agent that returns 1 for input [0,0,1,1] can be interpreted as accepting the structure where $B=\{o_{3},o_{4}\}$ (if 1 is interpreted as true in the structure and in the quantifier), as rejecting the structure where $B=\{o_{3},o_{4}\}$ (if 1 is interpreted as false in the quantifier and true in the structures), as accepting the structure where $B=\{o_{1},o_{2}\}$ (if 1 is interpreted as true in the quantifier and false in the structures), or as rejecting the structure where $B=\{o_{1},o_{2}\}$ (if 1 is interpreted as false in the quantifier and the structures). Crucially, the interpretation of the bits has to be consistent across the structures and, in a possibly different way, across the quantifier judgments. Therefore, each agent can be interpreted as speaking four quantifiers, depending on whether 1 and 0 are interpreted as meaning true or false in the structures and in the agent's output.

The issue of interpreting bits as Boolean values is important when measuring monotonicity, because the value of monotonicity depends on the attribution of truth values, rather than simply bits, to each structure, rather than simply to each binary string. Since no objective truth values or structures can be recovered from the network, in practice we measure the monotonicity of a neural network's language as the highest among the degrees of upward monotonicity of the four quantifiers compatible with the network's judgments for all possible structures. Selecting the highest among the degrees of monotonicity compatible with the neural networks' output allowed us to more easily identify, classify, and visualize the emerging patterns that we discussed in the Sections 3.2 and 4.2.

We discussed in Section 3.1.5 the problem of defining a measure of downward monotonicity based on its upward version. Having a separate measure of downward monotonicity turns out to be in fact superfluous when considering the data produced by the neural networks. Each agent can be interpreted as instantiating any of four quantifiers, depending on how the Booleans are interpreted for the quantifier's input and output. The two interpretations of the truth values of the structures—associating 0 with false and 1 with true, or vice versa—correspond to an exchange of the subset–superset relation of any two structures. For instance, the structure [0,1,1] with 0 interpreted as false and 1 interpreted as true is a superstructure of [0,0,1], but the former becomes a substructure of the latter when 0 is interpreted as true and 1 as false. It follows that the measure of downward monotonicity is equivalent to the measure of upward monotonicity with the opposite interpretation of the Booleans representing the structure. Therefore, the measure of upward monotonicity, when applied to all four possible interpretations of the network's output, also covers a possible interpretation of the network as instantiating a downward monotone quantifier.

## APPENDIX B Other optimizers

In the computational models above, we have used the *Adam* optimizer. However, it is worth considering how robust the results are across different optimizers. We check this for two other common optimizers, namely *stochastic gradient descent* (SGD) and SGD + momentum. Results show that although the exact proportions of the different types of quantifiers that emerge are different, the main effects are robust across different optimizers.

### B.1 Experiment 1 with other optimizers

We ran the model with SGD for the same combination of parameters as shown in Fig. 4. The results are shown in Fig. B1. As expected, languages get to high levels of monotonicity, although the precise levels are different than with the ADAM optimizer. For bottleneck of 200, about half or all evolved languages are ultrafilters. For eight epochs and bottlenecks greater than 200, about a quarter of all languages become degenerate. SGD + momentum again shows the same pattern (Fig. B2).

### B.2 Experiment 2 with other optimizers

We ran the model with shuffled inputs with SGD for the same combination of parameters as shown in Fig. 6. The results are shown in Fig. B3 for SGD and Fig. B4 for SGD + momentum. Like in the results from the experiment with ADAM (Fig. 6), the languages tend to divide in two clusters, a degenerate or close to degenerate cluster (yellow) and a high‐quantity high‐monotonicity cluster (top right of plots). One difference is that the proportion of degenerate languages is higher in the SGD + momentum model.
